# Supplementary material for: Standing Desks in a Grade 4 Classroom over the Full School Year
Source: Int J Environ Res Public Health. 2019 Sep 25;16(19):3590. doi: 10.3390/ijerph16193590 (PMC6801749; doi:10.3390/ijerph16193590)
Supplement: Supplementary file 1 [file ijerph-16-03590-s001.pdf]

Supplementary table:

Table S1. Sitting and standing time at the start and the end of the school year

	Start of school year	End of school year
**Standing desk**		
**Standing time** (mins/school day (95% confidence intervals)	81 (64 to 94)^	95 (84 to 107)^
**Sitting time** (mins/school day (95% confidence intervals)	211 (198 to 224)*	175 (164 to 187)*
**Traditional seated desk**		
**Standing time** (mins/school day (95% confidence intervals)	64 (53 to 76)^	69 (58 to 81)^
**Sitting time** (mins/school day (95% confidence intervals)	228 (217 to 239)*	215 (203 to 226)*

* Significant reduction in sitting time when using a standing desk at both time points

^ Significant increase in standing time when using a standing desk at both time points
